# Preventing false discovery of heterogeneous treatment effect subgroups in randomized trials

**DOI:** 10.1186/s13063-018-2774-5

**Published:** 2018-07-16

**Authors:** Joseph Rigdon, Michael Baiocchi, Sanjay Basu

**Affiliations:** 1 Quantitative Sciences Unit, Stanford University School of Medicine, 1070 Arastradero Road #3C3104, MC 5559, Palo Alto, California 94304 USA; 2 Stanford Prevention Research Center, Stanford University School of Medicine, Medical School Office Building, Room 318,1265 Welch Road, MC 5411, Stanford, CA 94305 USA; 3 Departments of Medicine and of Health Research and Policy, Center for Primary Care and Outcomes Research and Center for Population Health Sciences, Stanford University School of Medicine, 1070 Arastradero Road, Office 282 MC 5560, Palo Alto, CA 94304 USA

**Keywords:** Classification and regression trees, Decision support tool, Heterogeneous treatment effects, Matching

## Abstract

**Background:**

Heterogeneous treatment effects (HTEs), or systematic differences in treatment effectiveness among participants with different observable features, may be important when applying trial results to clinical practice. Current methods suffer from a potential for false detection of HTEs due to imbalances in covariates between candidate subgroups.

**Methods:**

We introduce a new method, matching plus classification and regression trees (mCART), that yields balance in covariates in identified HTE subgroups. We compared mCART to a classical method (logistic regression [LR] with backwards covariate selection using the Akaike information criterion ) and two machine-learning approaches increasingly applied to HTE detection (random forest [RF] and gradient RF) in simulations with a binary outcome with known HTE subgroups. We considered an *N* = 200 phase II oncology trial where there were either no HTEs (1A) or two HTE subgroups (1B) and an *N* = 6000 phase III cardiovascular disease trial where there were either no HTEs (2A) or four HTE subgroups (2B). Additionally, we considered an *N* = 6000 phase III cardiovascular disease trial where there was no average treatment effect but there were four HTE subgroups (2C).

**Results:**

In simulations 1A and 2A (no HTEs), mCART did not identify any HTE subgroups, whereas LR found 2 and 448, RF 5 and 2, and gradient RF 5 and 24, respectively (all false positives). In simulation 1B, mCART failed to identify the two true HTE subgroups whereas LR found 4, RF 6, and gradient RF 10 (half or more of which were false positives). In simulations 2B and 2C, mCART captured the four true HTE subgroups, whereas the other methods found only false positives.

All HTE subgroups identified by mCART had acceptable treated vs. control covariate balance with absolute standardized differences less than 0.2, whereas the absolute standardized differences for the other methods typically exceeded 0.2. The imbalance in covariates in identified subgroups for LR, RF, and gradient RF indicates the false HTE detection may have been due to confounding.

**Conclusions:**

Covariate imbalances may be producing false positives in subgroup analyses. mCART could be a useful tool to help prevent the false discovery of HTE subgroups in secondary analyses of randomized trial data.

## Background

Precision medicine aims to direct the right medication to the right patient at the right time [1]. A requirement of precision medicine is that the effect of a treatment on a given patient must be accurately estimated, to determine if that patient systematically differs from the average in a randomized trial, for instance.

Heterogeneous treatment effects (HTEs)—or systematic differences in treatment effects among participants with different observable features—are increasingly identified through post-trial data analyses [2, 3]. Because common univariate subgroup analyses of randomized trial data have been found to lack statistical power to detect HTEs [4], some researchers have deployed multivariate models to detect how covariates may produce different expected treatment effects for different participants based on a multivariate risk/benefit score [5]. Both traditional statistical regression approaches [6] and newer machine-learning methods such as random forest (RF) methods [7] are increasingly being used to estimate HTEs.

HTE estimation approaches attempt to predict individual-level treatment effects and identify subgroups of participants with above average or lower average benefit from a treatment. In this paper, we focus on identifying subgroups with HTE, rather than individual-level treatment effects. A major challenge in HTE subgroup analysis is that, even if the data come from a randomized trial, subgroups that are identified may be imbalanced on important clinical characteristics [8]. For example, for a blood pressure treatment trial, one subgroup with high benefit from treatment may be individuals of black race with baseline systolic blood pressure >140 mmHg. Yet it may be that among individuals with black race and baseline systolic blood pressure >140 mmHg, those randomized to the treatment arm had much better kidney function than those randomized to the control arm. Hence, race and baseline blood pressure may not have correctly identified a HTE, since the subgroup identified may have been falsely detected due to an imbalance in another feature (kidney function) between the treatment and control groups. While randomization aims to avoid such confounding in the overall trial, it does not guarantee that all subgroups of interest will achieve adequate balance to avoid confounding in the estimation of a subgroup-specific effect. If HTE subgroups were known in advance, restricted or constrained randomization within subgroups would be an attractive option for inference on the HTEs. However, in practice, HTE subgroups are rarely known in advance and creating subgroups from combinations of all levels of prognostic categorical variables (such as those listed in Table 1) would likely lead to an impractical randomization scheme.

**Table 1:** Overview of study designs considered in simulations

Effect type	Study setting	
	*N* = 200 phase II oncology trial	*N* = 6000 phase III CVD trial
ATE > 0, no HTE subgroups	Simulation 1A	Simulation 2A
ATE> 0, HTE subgroups exist	Simulation 1B	Simulation 2B
ATE = 0, HTE subgroups exist		Simulation 2C

*ATE* average treatment effect, *CVD* cardiovascular disease, *HTE* heterogeneous treatment effect

The consequences of falsely identifying a subgroup can be dire: a life-saving treatment could be withheld or an ineffective treatment prescribed that increases the chances of a serious adverse event. One potential strategy to reduce the likelihood of false HTE detection is to reduce the observed imbalance between treatment and control groups when applying methods to detect HTE [8].

Here, we propose a method for detecting and potentially avoiding imbalance in observed characteristics in trial data when constructing HTE models for precision medicine applications. We specifically combine matching (to control for treated vs. control covariate imbalances) with classification and regression trees (to identify subgroups with an easily visualized decision tree), and demonstrate the virtues of this approach compared to popular alternatives currently implemented in the post-hoc trial analysis literature.

## Methods

We simulated multiple trials in which the true HTEs were known, to compare the rates of finding true and false positive subgroups from: (i) the matching with classification and regression trees approach (mCART), (ii) a logistic regression (LR) model with interaction terms between participant covariates and the treatment arm, (iii) the common machine-learning approach of RF analysis, and (iv) the newer machine-learning approach specifically designed for detecting HTEs, of gradient forest analysis (sometimes referred to as causal forest analysis, a term we intentionally avoid here [9]). These methods are detailed further below. Statistical code for replication is available at https://github.com/joerigdon/HTE.

### Simulated datasets

We simulated trial data to test the ability of comparator methods to detect HTEs. We simulated randomized trials in which each individual participant has a potential adverse medical event when randomized to treatment, *Y*_1_, or when randomized to placebo, *Y*_*0*_. In practice, only one of *Y*_1_ or *Y*_*0*_ is revealed by the trial [10]. The outcome variable is labeled 0 if no event occurs and 1 if an event occurs. The unobservable true treatment effect for each individual in the trial, *δ* = *Y*_1_ – *Y*_0_, is the difference between the outcome for the treatment group and the outcome for the control group. *δ* = −1 if the treatment prevents the event (benefit), *δ* = 0 if the treatment has no effect on the event, and *δ* = 1 if the treatment induces the event (harm).

We considered two study settings (outlined in Table 1) representative of clinical trials often seen in practice: (1) a smaller phase II oncology trial (*N* = 200) and (2) a larger phase III trial of a cardiovascular disease (CVD) treatment (*N* = 6000). In setting (1), we considered a trial where researchers were interested in replicating the finding that a combination of treatments was more effective than a single treatment in increasing progression-free survival among patients with advanced, estrogen receptor-positive, HER2-negative breast cancer [11]. We designed the study to have 80% power to detect a change from an event rate of 59/81 (about 73%) in the single treatment group (*Y*_0_) to an event rate of 41/84 (about 49%) in the combination group (*Y*_1_). Such a study design would require *n* = 64 per group [12], but given the high number of anticipated dropouts and conservative effect-size estimation (73% rounded to 70% and 49% to 50%), we recruited *n* = 100 per group in the hypothetical trial.

Let *V* ~ N(*m*, *s*) be shorthand for variable *V* is normally distributed with mean *m* and standard deviation *s.* Let *V* ~ Bern(*b*) be shorthand for variable *V* is drawn from a Bernoulli distribution with mean *b*, or Pr[*V* = 1] = *b*, and let *V* ~ Multinom(*p*_*A*_, *p*_*B*_, …, *p*_*Z*_) be shorthand for *V* is drawn from a multinomial distribution where *V* can take on the values (*A*, *B*, …, *Z*) with corresponding probabilities (*p*_*A*_, *p*_*B*_, …, *p*_*Z*_). For setting (1), the oncology trial, we simulated *n* = 200 records of the following six baseline covariates: age ~ N(65, 5), disease stage = 4 ~ B(0.98), disease site = (visceral, bone only, other) ~ Multinom(0.5, 0.17, 0.33), previous treatment = (none, chemo only, hormonal only, chemo + hormonal) ~ Multinom(0.5, 0.2, 0.2, 0.1), Eastern Cooperative Oncology Group (ECOG) score ~ Bern(0.55), and disease-free interval >12 months from adjuvant to recurrence ~ Bern(0.35).

We simulated the treatment effects in two ways for the oncology trial. In simulation 1A, we considered a setting where there were no HTE subgroups, i.e., *δ* = (–1, 0, 1) *~* Multinom(0.5, 0.2, 0.3) for all *N* = 200 individuals in the trial, independent of covariates such that the average treatment effect was approximately –0.2. For individuals with δ = −1, *Y*_1_ and *Y*_0_ immediately follow as 0 and 1, respectively, and for individuals with δ = 1, *Y*_1_ and *Y*_0_ immediately follow as 1 and 0, respectively. For individuals with δ = 0, *Y*_1_ and *Y*_0_ were set to 1, so that our sample means were approximately *Y*_1_ = 0.5 and *Y*_0_ = 0.7.

In simulation 1B, we considered a setting where there were HTE subgroups in the oncology trial in two groups: women over 65 years old versus women 65 years and younger, each constituting about half of the sample population of the trial. In particular, for women aged ≤65, *δ ~* Multinom(0.6, 0.2, 0.2), such that the average treatment effect (ATE) was approximately −0.4 (*Y*_1_ = 0.4 and *Y*_0_ = 0.8), and for women aged >65, *δ ~* Multinom(0.4, 0.2, 0.4), such that the ATE was approximately 0 (*Y*_1_ = 0.6 and *Y*_0_ = 0.6), and such that the overall ATE was still approximately −0.2.

In setting (2), we considered a trial where researchers were interested in testing the effect of a more intensive blood pressure target of systolic pressure <20 mmHg versus the standard target for systolic pressure <140 mmHg for preventing a composite CVD outcome [13]. We designed the study to have 80% power to detect a change from an event rate of 6.8% in the standard (*Y*_0_) to an event rate of 5.2% in the intensive target group (*Y*_1_). Such a study design would require *n* = 3000 per group [12] to have 80% power to reject the null hypothesis that standard and intensive are equal versus the alternative specified above (6.8% standard versus 5.2% intensive).

For setting (2), the CVD trial, we simulated *n* = 6000 records of the following 10 baseline covariates: age ~ N(68, 10), black race ~ B(0.3), baseline systolic blood pressure (mm Hg) ~ N(140, 15), baseline diastolic blood pressure (mm Hg) ~ N(78, 12), serum creatinine (mg/dl) ~ N(1.07, 0.34), estimated glomerular filtration rate (eGFR; ml/min/1.73 m^2^) ~ N(72, 20), statin use ~ B(0.43), aspirin use ~ B(0.51), Framingham 10-year risk score for a CVD event ~ N(25, 12), and smoking status = (never, former, current) ~ Multinom(0.44, 0.42, 0.14).

In simulation 2A, we considered a setting where there was an ATE of approximately *D* = −1.6% (−0.016) in the CVD trial, but no HTE subgroups. In particular, *δ* = (−1, 0, 1) *~* Multinom(0.068, 0.88, 0.052) for all *N* = 6000 individuals in the trial independent of covariates, such that the ATE was approximately −0.016 (*Y*_1_ = 0.052 and *Y*_0_ = 0.068).

In setting 2B, we simulated HTEs by setting:

(i) For individuals taking aspirin with eGFR ≤ 72, *δ ~* Multinom(0.06, 0.88, 0.06) such that Δ = 0 × *D* = 0.
(ii) For individuals taking aspirin with eGFR > 72, *δ ~* Multinom(0.012, 0.88, 0.108) such that Δ = −6 × *D* = 0.096.
(iii) For individuals not taking aspirin with eGFR ≤ 72, *δ ~* Multinom(0.116, 0.88, 0.004) such that Δ = 7 × *D* = −0.112.
(iv) For individuals not taking aspirin with eGFR > 72, *δ ~* Multinom(0.084, 0.88, 0.036) such that Δ = 3 × *D* = −0.048.

The overall ATE was approximately Δ = *D* × (0 – 6 + 7 + 3) / 4 = *D* = −0.016 (*Y*_1_ = 0.052 and *Y*_0_ = 0.068).

In simulation 2C, we consider the same *N* = 6000 CVD trial where ATE is 0 but there are HTE subgroups. In particular:

(i) For individuals taking aspirin with eGFR ≤72, *δ ~* Multinom(0.036, 0.88, 0.084) such that Δ = −3 × *D* = 0.048.
(ii) For individuals taking aspirin with eGFR > 72, *δ ~* Multinom(0.004, 0.88, 0.116) such that Δ = −7 × *D* = 0.112.
(iii) For individuals not taking aspirin with eGFR ≤72, *δ ~* Multinom(0.116, 0.88, 0.004) such that Δ = 7 × *D* = −0.112.
(iv) For individuals not taking aspirin with eGFR > 72, *δ ~* Multinom(0.084, 0.88, 0.036) such that Δ = 3 × *D* = − 0.048.

The overall ATE was approximately Δ = *D* × (−3 – 10 + 7 + 3) / 4 = 0 × *D* = 0.

### mCART methodological approach

To identify HTE subgroups that are balanced on the covariates, we propose a novel algorithm using rank-based Mahalanobis distance matrix matching followed by classification trees for inference on the HTEs. Henceforth, we term this method matching plus classification and regression trees (mCART):

1. We select a set of *K* prognostic variables of interest, *P* = (*X*_1_, …, *X*_*K*_), where practice or literature suggests a potential effect modification in *δ*, e.g., age, sex, race, medical history, etc. These *K* variables are often selected beforehand by research teams (e.g., the demographic and risk factors displayed in a typical Table 1) and can be continuous or categorical.
2. For each participant i = 1, ..., N in the randomized trial, the covariate vector *P*_*i*_ = (*x*_*i*1_, …, *x*_*iK*_) is collected and stored.
3. Suppose there are *C* individuals randomized to the control group and *T* to the treatment group (such that *C + T* = *N*). Then, a rank-based Mahalanobis distance matrix with *T* rows (treated individuals) and *C* columns (control individuals) is formed. If *C > T* the matrix is transposed.
4. A pair-matching algorithm [14] is applied to the matrix in step 3 to create *G* = min(*C*, *T*) pair matches, each containing one treated individual and one control individual.
5. For match *g* = 1, …, *G*, the covariate vectors *P*_*g*_^*t*^ (treated) and *P*_*g*_^*c*^ (control) are compared. If for match *g*, any of the categorical variables, e.g., race or sex, in *P*_*g*_^*t*^ are unequal to their counterpart in *P*_*g*_^*t*^, then match *g* is discarded from the set of *G* matches. After this step, there are *G*_2_ ≤ *G* matches remaining. We do not anticipate losing an impactful number of matches as mCART is designed for settings where there are 10–15 prognostic variables of interest to be balanced at baseline (shown in Table 1), of which perhaps 7–10 are categorical.
6. We use the *G*_2_ matched pairs to create an averaged data set as follows. For match *g* = 1, …, *G*_2_, *δ*_*g*_ = *Y*_g_^t^ – *Y*_g_^c^ ∈{−1, 0, 1}, and the vector of covariates *P*_*g*_ is equal to (*P*_*g*_^*t*^ + *P*_*g*_^*c*^) / 2, the average of the treated and control participants in pair *g*.
7. We apply a single conditional inference tree [15] to model *δ*_*g*_ as a function of *P*_*g*_ in the averaged data set from step 6. This yields a decision tree that estimates where there are differences in the distribution of *δ*_*g*_, i.e., where there are heterogeneous treatment effects. By virtue of the match, any identified subgroups have an approximately equal distribution of risk factors between the treated individuals and controls. The tree will split important categorical variables by level and continuous variables by cut points.
8. We apply the model estimated in step 7 to our *N* individual by *K* variable observed data collected in the randomized trial to estimate *δ*_*i*_, Pr[*δ*_*i*_ = − 1], Pr[*δ*_*i*_ = 0], and Pr[*δ*_*i*_ = 1] for *i* = *1*, …, *n.* Within the terminal nodes identified in step 7, we can estimate the ATE in the original data set using methods for inference on a risk difference.

### Comparison methods

We compared our method to three strategies commonly applied or proposed for identifying HTEs: LR, RF, and gradient RF. Unless otherwise specified, default settings for parameters in RF or gradient RF were used.

In LR, all variables, treatments, and the interaction of treatment with each of the individual variables were entered into the model. The backwards Akaike information criterion was used to select the most parsimonious model, as is typical in the literature for HTE detection [16, 17]. After obtaining the final model, a probability of the outcome for the treatment group, *p*_1_, and a probability of the outcome for the control group, *p*_0_, were estimated for each of the 6000 individuals. Treatment effects were estimated for each individual as *p*_1_ – *p*_0_. Estimated treatment effects were partitioned into subgroups using one classification and regression tree (via the R package ‘party’) [15].

In the RF method, all variables and a treatment dummy variable were entered into the model. The RF method searches across all available variables to find the first variable that explains the largest variance in the outcome, and it chooses a value of that variable to split the population into subgroups. Then, a second variable is chosen, then a third, producing a tree where the branches identify subgroups. The process is repeated hundreds of times with bootstrapped samples of the data and covariates, to produce a forest of these trees, and the predicted outcome for an individual is taken as the average prediction from among the trees in the forest [18]. We did not specifically enter any interaction terms because the RF method searches for interactions by construction. The RF algorithm was applied by taking 500 bootstrap samples with replacement of the data. The best split at each node in each tree is chosen among a randomly sampled group of the square root of the total number of variables. The R package ‘randomForest’ was used to for the modeling [19]. A predicted outcome (equal to 0 or 1) was obtained for each individual as the most common prediction of 0 or 1 from the 500 trees, for both the treatment group and the control group. The difference in predicted outcomes served as each individual’s treatment effect estimate. Estimated treatment effects (−1, 0, or 1 for each individual) were again partitioned into subgroups using one classification and regression tree in the R package ‘party’.

In the gradient RF method, the RF is built to yield an estimate in the interval [−1, 1] for *δ* for each individual. A key difference between the gradient RF method and the RF method is the process known as honest estimation, which means that the gradient RF approach selects the variables defining each split point/branch of the decision tree from one subset of the data, then estimates the values of each variable that define the split in a different subset, to reduce the bias in treatment effect estimation and the influence of outliers [9]. Additionally, the gradient RF algorithm applies a classification tree to each of 2000 bootstrap samples. It (i) finds terminal nodes of individuals with similar covariates (*X*) and (ii) computes an effect estimate within each node as the proportion experiencing the outcome in the treated group minus the proportion experiencing the outcome in the control subset of the trial. Using similar logic as the RF method, the predicted treatment effects are averaged across the 2000 trees to yield an estimated causal effect for each individual in the trial. Gradient RF was carried out using the R package ‘grf’ [20]. After building the risk model, estimates of *δ* were again partitioned into subgroups using a classification tree.

Simulations were performed in R [21], using the simulation code posted at https://github.com/joerigdon/HTE. In simulations 1A, 1B, 2A, 2B, and 2C, one hypothetical trial was generated by assigning half of the simulated participants to the treatment group (*Z* = 1) and half to the control group (*Z* = 0), and the outcome for each individual was calculated as *Y* = *Z* × *Y*_1_ + (1 – *Z*) × *Y*_0_. For all methods, after subgroup identification, HTEs were computed within identified subgroups along with treated minus control absolute standardized differences (ASDs) for every covariate. Subgroups were deemed to have acceptable balance if all ASDs were below 0.2, a cutoff point chosen because it is of the same order as small effect sizes [22]. Bias was also computed within each subgroup as the estimated ATE for the subgroup. Bias is the difference in proportions of the outcome for treated individuals minus controls in the subgroup, minus the true ATE for the subgroup (known as the average of the true *δ*’s in the subgroup).

## Results

### Simulation 1A

The characteristics of the simulated trial population for simulation 1A are displayed in Table 2. It shows that the important covariates in the simulated trial were balanced across the overall treated and control groups, indicating successful randomization, and thus allowing average differences in outcomes (*Y*) to be attributed to control or treatment exposure without concerns due to confounding arising from baseline covariates. The outcomes were: 46/100 individuals in the combination group experienced the event, while 70/100 individuals in the single agent arm experienced the event. The risk difference was $\widehat{\Delta}$ = –0.24 [95% confidence interval (0.38, –0.01)], which is close to the true value Δ = –0.2. The treatment was effective in the sense that the upper limit of the confidence interval of the treatment effect was less than 0, indicating a decrease in the number of deaths for the combination treatment group.

**Table 2:** Data at randomization for simulation 1A

	Single agent	Combination	ASD
	*n* = 100	*n* = 100	
Age (years)	65.0 (±5.8)	64.9 (±5.0)	0.01
Stage 4			
No	4 (4.0%)	1 (1.0%)	0.19
Yes	96 (96.0%)	99 (99.0%)	0.19
Site			
Visceral	53 (53.0%)	49 (49.0%)	0.08
Bone only	13 (13.0%)	13 (13.0%)	0
Other	34 (34.0%)	38 (38.0%)	0.08
Previous treatment			
None	56 (56.0%)	52 (52.0%)	0.08
Chemo only	17 (17.0%)	19 (19.0%)	0.05
Hormonal only	16 (16.0%)	19 (19.0%)	0.08
Chemo + hormonal	11 (11.0%)	10 (10.0%)	0.03
ECOG score			
0	45 (45.0%)	37 (37.0%)	0.16
1	55 (55.0%)	63 (63.0%)	0.16
Disease free > 12 months adjuvant to recurrence			
No	65 (65.0%)	62 (62.0%)	0.06
Yes	35 (35.0%)	38 (38.0%)	0.06

*ASD* absolute standardized difference, *ECOG* Eastern Cooperative Oncology Group

**Fig. 1:**
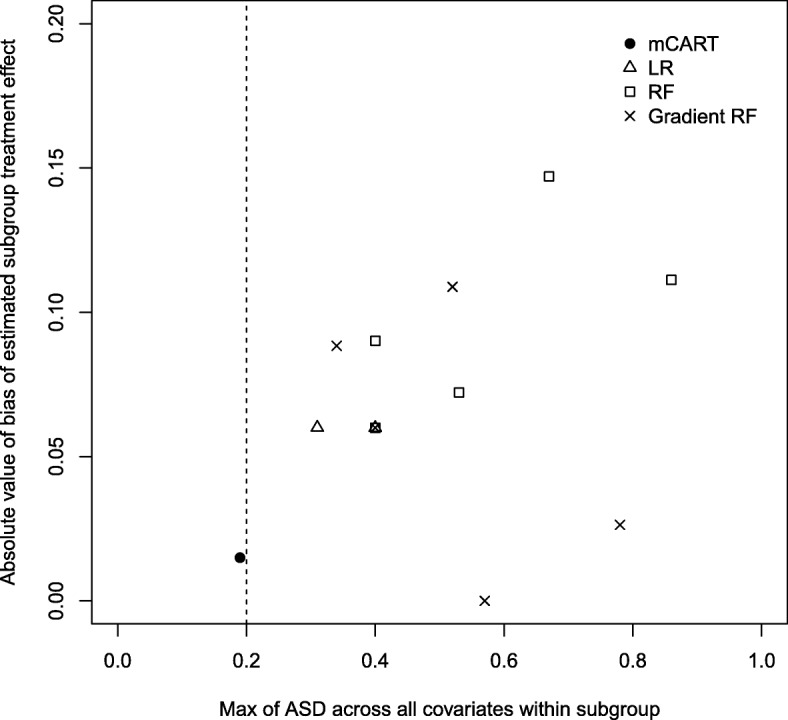
Plot of maximum absolute standardized difference (ASD) within node for each method (*x*-axis) versus absolute bias (absolute value of estimated treatment effect minus true treatment effect) in each identified node for LR, RF, gradient RF, and mCART in simulation 1A. All identified subgroups to the left of the vertical dashed line of 0.2 have an acceptable balance. ASD absolute standardized difference, LR logistic regression, mCART matching plus classification and regression trees, RF random forest

In subgroup analyses of the data shown in Table 2, mCART did not find any subgroups (no false positives), whereas LR found 2, RF 5, and gradient RF also 5 (all false positives). Tables 4, 5 and 6 in the appendix show the balance statistics for the discovered subgroups for LR, RF, and gradient RF, respectively. Figure 1 displays a plot of the maximum ASD within subgroup versus the bias for each of the methods.

### Simulation 1B

Table 7 in the appendix shows the data at baseline for simulation 1B. Data are balanced across the single agent and combination groups with the minor exception of previous treatment equals none (ASD = 0.22; note that ASD < 0.2 is the rule of thumb for small effect-size differences or an acceptable balance [22]). The trial is a success as the combination group (29/100) is shown to have a lower event rate than the single-agent group (56/100) with a risk difference of −0.27 (−0.41, −0.13).

Simulation 1B contained two subgroups with HTEs: women ≤65 years of age and women >65. In the subgroup analyses, mCART did not find any subgroups (two false negatives), whereas LR found 4, RF 6, and gradient RF also 10 (all false positives). Fig. 4 in the appendix is a plot of the maximum ASD within subgroup versus the bias for each of the methods.

### Simulation 2A

Table 3 shows the data at baseline for simulation 2A. Data are balanced across the treatment and placebo groups with all ASDs < 0.2. The trial is a success as the treatment group (157/3000) is shown to have a lower event rate than the control group (203/3000) with a risk difference of −0.015 (−0.028, −0.0030).

**Table 3:** Data at randomization for simulation 2A

	Placebo	Treatment	ASD
	*n* = 3000	*n* = 3000	
Age (years)	68.1 (±10.1)	68.1 (±10.0)	0
Black race			
No	2133 (71.1%)	2075 (69.2%)	0.04
Yes	867 (28.9%)	925 (30.8%)	0.04
Systolic blood pressure (mm Hg)	140.8 (±15.1)	140.1 (±15.1)	0.05
Diastolic blood pressure (mm Hg)	78.1 (±11.8)	77.7 (±11.9)	0.04
Serum creatinine (mg/dl)	1.1 (±0.3)	1.1 (±0.3)	0.06
Estimated GFR (ml/min/1.73 m^2^)	72.2 (±19.6)	72.0 (±20.0)	0.01
Statin use			
No	1706 (56.9%)	1745 (58.2%)	0.03
Yes	1294 (43.1%)	1255 (41.8%)	0.03
Aspirin use			
No	1446 (48.2%)	1410 (47.0%)	0.02
Yes	1554 (51.8%)	1590 (53.0%)	0.02
Framingham risk score	25.2 (±12.2)	25.2 (±11.9)	0
Smoking status			
Never	1350 (45.0%)	1348 (44.9%)	0
Former	1244 (41.5%)	1220 (40.7%)	0.02
Current	406 (13.5%)	432 (14.4%)	0.03

*ASD* absolute standardized difference, *GFR* glomerular filtration rate

Simulation 2A contained no subgroups with HTEs. In subgroup analyses, mCART did not find any subgroups, whereas LR found 448, RF 2, and gradient RF also 24 (all false positives). Fig. 5 in the appendix is a plot of the maximum ASD within subgroup versus the bias for each of the methods.

### Simulation 2B

Table 8 in the appendix shows the data at baseline for simulation 2B. Data are balanced across the treatment and placebo groups with all ASDs< 0.2. The trial is a success as the treatment group (160/3000) is shown to have a lower event rate than the control group (203/3000) with a risk difference of −0.014 (−0.027, −0.0019).

**Fig. 2:**
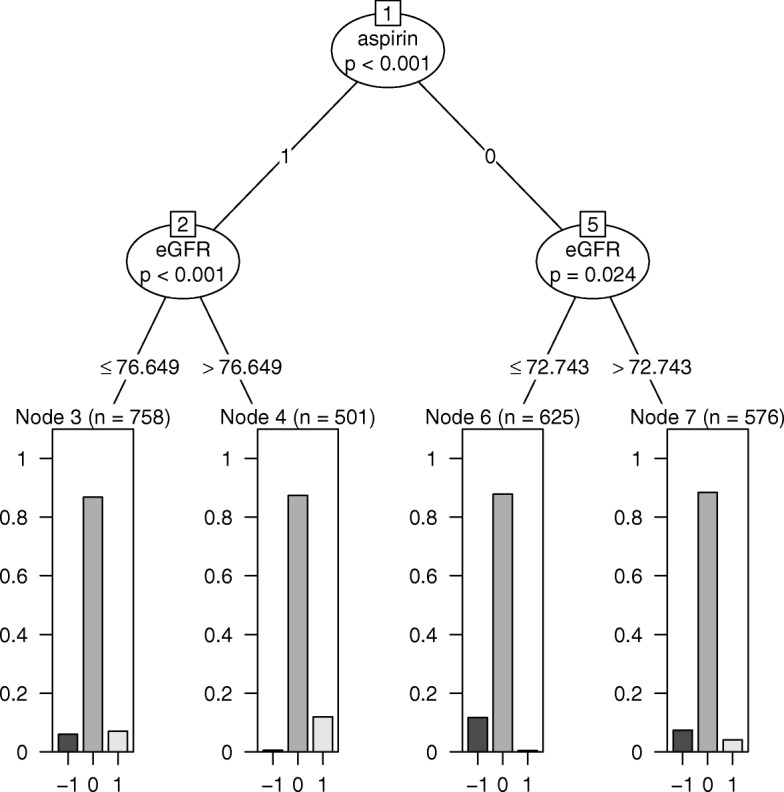
mCART results from simulation 2B. eGFR estimated glomerular filtration rate, mCART matching plus classification and regression trees

**Fig. 3:**
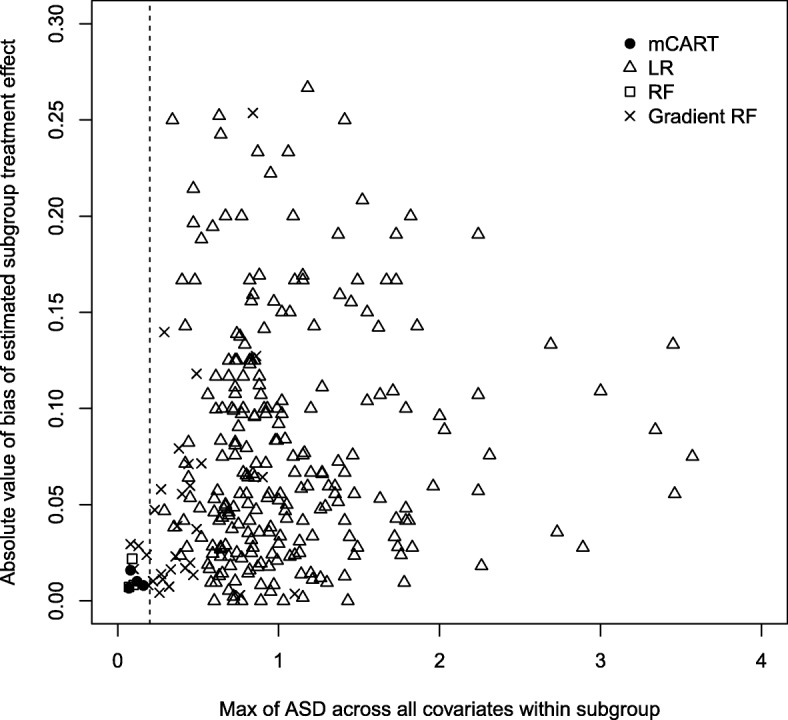
Plot of maximum absolute standardized difference (ASD) within node for each method (*x*-axis) versus absolute bias (absolute value of estimated treatment effect minus true treatment effect) in each identified node for LR, RF, gradient RF, and mCART in simulation 2B. All identified subgroups falling to the left of the vertical dashed line of 0.2 have an acceptable balance. ASD absolute standardized difference, LR logistic regression, mCART matching plus classification and regression trees, RF random forest

**Fig. 4:**
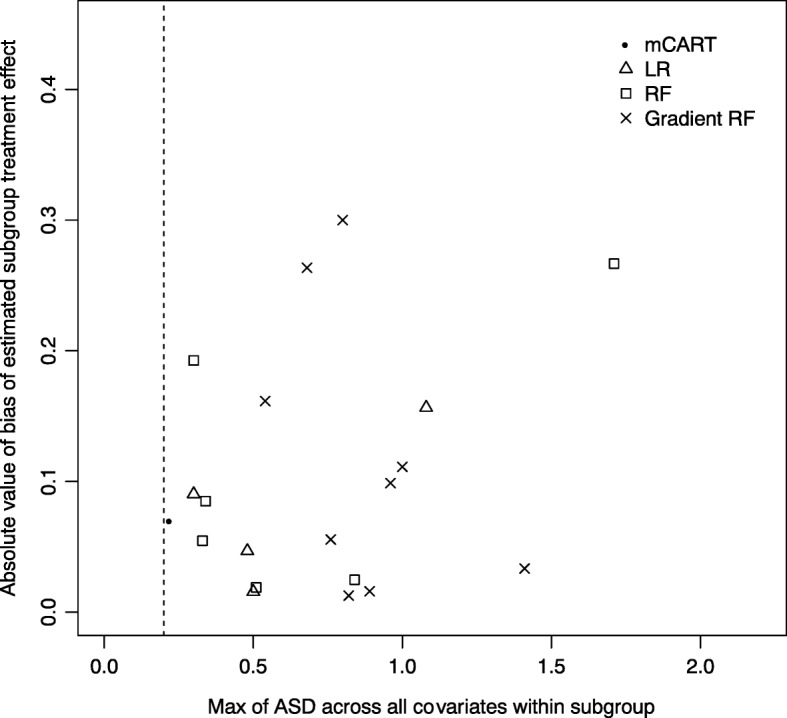
Plot of maximum absolute standardized difference (ASD) within node for each method (*x*-axis) versus absolute bias (absolute value of estimated treatment effect minus true treatment effect) in each identified node for LR, RF, gradient RF, and mCART in simulation 1B. All identified subgroups falling to the left of the vertical dashed line of 0.2 have an acceptable balance. ASD absolute standardized difference, LR logistic regression, mCART matching plus classification and regression trees, RF random forest

**Fig. 5:**
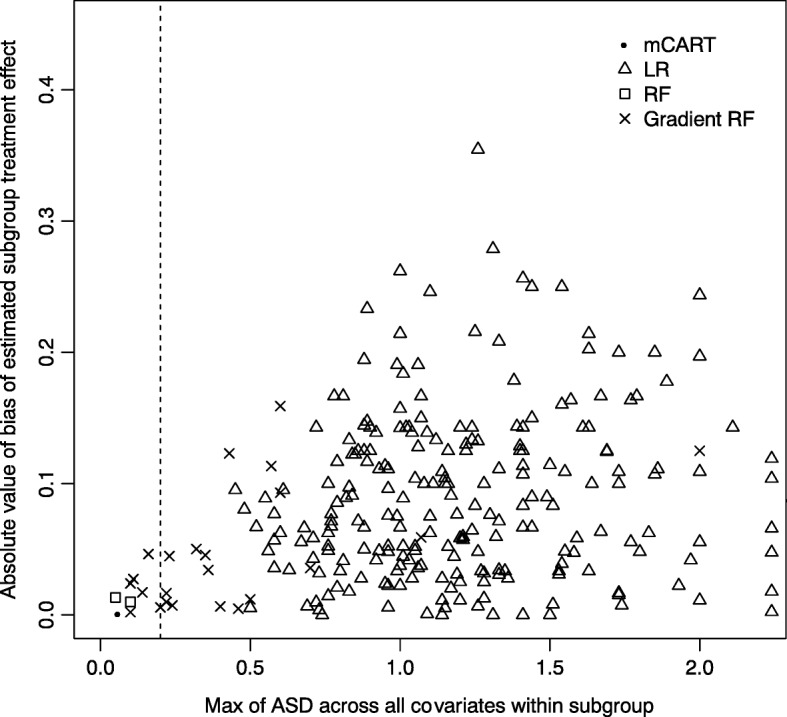
Plot of maximum absolute standardized difference (ASD) within node for each method (*x*-axis) versus absolute bias (absolute value of estimated treatment effect minus true treatment effect) in each identified node for LR, RF, gradient RF, and mCART in simulation 2A. All identified subgroups falling to the left of the vertical dashed line of 0.2 have an acceptable balance. ASD absolute standardized difference, LR logistic regression, mCART matching plus classification and regression trees, RF random forest

Simulation 2B contained four subgroups with HTEs as outlined in ‘Methods’: individuals taking aspirin with eGFR ≤ 72, individuals taking aspirin with eGFR > 72, individuals not taking aspirin with eGFR ≤ 72, and individuals not taking aspirin with eGFR > 72. In the subgroup analyses, mCART found four subgroups (see Fig. 2), whereas LR found 436, RF 3, and gradient RF 37 (all false positives). The four subgroups found by mCART approximately equaled the four true subgroups: individuals taking aspirin with eGFR ≤ 76.649 (versus 72), individuals taking aspirin with eGFR > 76.649 (versus 72), individuals not taking aspirin with eGFR ≤ 72.743 (versus 72), and individuals not taking aspirin with eGFR > 72.743 (versus 72). Figure 3 is a plot of the maximum ASD within subgroup versus the bias for each of the methods. Notably, all the subgroups discovered by mCART had maximum ASDs < 0.2 and bias never exceeding 0.016.

### Simulation 2C

Simulation 2C had no ATE but the same four HTE subgroups as in simulation 2B: individuals taking aspirin with eGFR ≤ 72, individuals taking aspirin with eGFR > 72, individuals not taking aspirin with eGFR ≤ 72, and individuals not taking aspirin with eGFR > 72. Table 9 in the appendix displays the study characteristics at baseline. The trial has a null result as the treatment group (193 / 3000) is shown to have the same event rate as the control group (190 / 3000) with a risk difference of 0.001 (−0.012, 0.014).

**Fig. 6:**
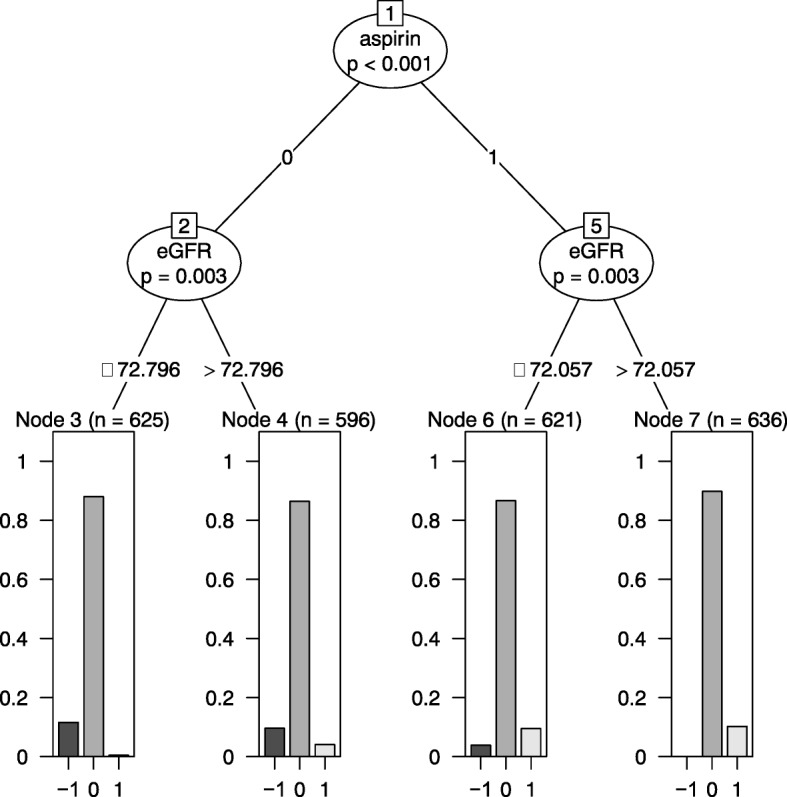
mCART results from simulation 2C. mCART matching plus classification and regression trees

**Fig. 7:**
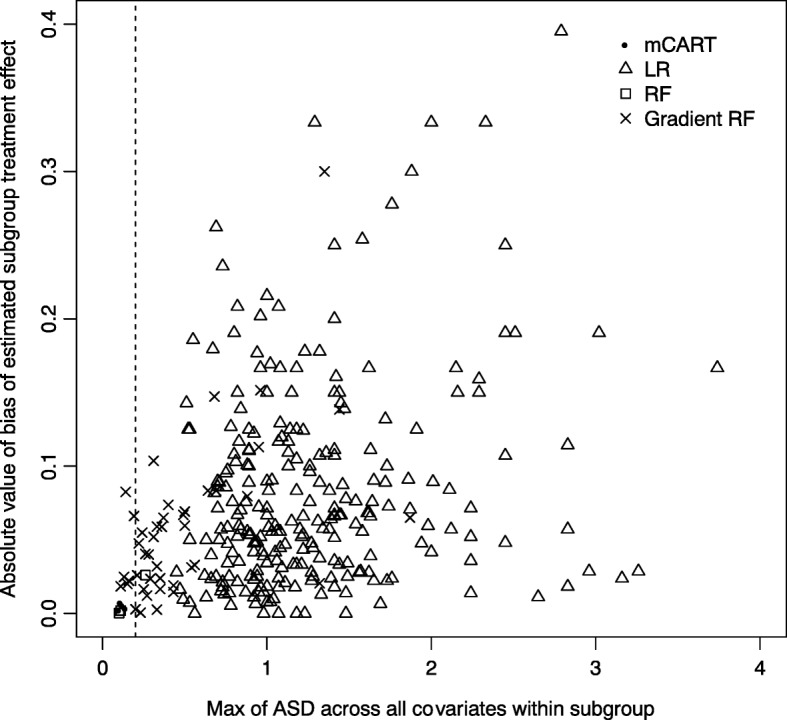
Plot of maximum absolute standardized difference (ASD) within node for each method (*x*-axis) versus absolute bias (absolute value of estimated treatment effect minus true treatment effect) in each identified node for LR, RF, gradient RF, and mCART in simulation 2C. All identified subgroups falling to the left of the vertical dashed line of 0.2 have an acceptable balance. ASD absolute standardized difference, LR logistic regression, mCART matching plus classification and regression trees, RF random forest

In the subgroup analyses, mCART found four subgroups (Fig. 6 in the appendix), whereas LR found 442, RF 3, and gradient RF 46 (all false positives). The four subgroups found by mCART approximately equaled the four true subgroups: individuals taking aspirin with eGFR ≤ 72.057 (versus 72), individuals taking aspirin with eGFR > 72.057 (versus 72), individuals not taking aspirin with eGFR ≤ 72.796 (versus 72), and individuals not taking aspirin with eGFR > 72.796 (versus 72). Fig. 7 in the appendix is a plot of the maximum ASD within subgroup versus the bias for each of the methods. Notably, all the subgroups discovered by mCART had maximum ASDs < 0.2 and bias never exceeding 0.008.

## Discussion

Precision medicine requires the detection of HTEs from randomized trial data to provide personalized effect estimates—that is, to determine if a particular patient is likely to experience benefits, no effects, or harms from therapy. This contextualizes the ATE in a trial for the individual patient.

Here, we found that while common standard regression and alternative machine-learning methods can identify HTE subgroups, they may also yield imbalances between the study arms within identified subgroups, such that differences in outcomes are falsely attributed to differences in treatment effect, but are in fact due to imbalances in covariates. We strongly recommend that researchers report the balance between study arms in identified subgroups to reduce the risk of false HTE reporting. Guidelines already recommend that studies estimating causal effects have a detailed discussion of the covariate balance of the groups under discussion [23, 24].

We also tested the method of matching followed by CART analysis and found it may reduce the imbalance in observable covariates and thereby prevent false HTE detection. The method yielded subgroups with a balance in observable characteristics, suggesting that differences in outcomes in identified subgroups were attributable to treatment or unobserved covariates. The method also produced an interpretable decision tree that may be more transparent to clinicians than alternative machine learning methods.

A limitation of our study is that we considered a handful of simple data-generating processes that we believe to be representative of trials hypothetically seen in clinical practice, with the intention of demonstrating a situation where an imbalance in subgroups can cause confounding. We do not know how often this will occur in practice, as this requires a further systematic review of the literature. Importantly, a second limitation is that real data could have unmeasured confounders that we cannot control for.

In future work, we hope to study how the choice of different matching algorithms impacts the performance of the mCART algorithm. Other avenues of future research include applying mCART to non-binary outcomes (e.g., continuous or survival outcomes), considering methods of inference for smaller sample sizes within identified subgroups (e.g., [25]), and further optimizing mCART for smaller trials (as it did not detect the two subgroups in the *N* = 200 oncology trial).

## Conclusions

mCART could be an interpretable and rigorous tool for identifying HTE subgroups after the conclusion of a clinical trial, and may help identify subgroups balanced on potential prognostic baseline variables that also differ in treatment effects. Perhaps most importantly, mCART may help prevent the wasteful false discovery of HTE subgroups in secondary analyses of randomized trials.

## Appendix

**Table 4:** Simulation 1A subgroup discovery using logistic regression (two subgroups found)

	Single agent	Combination	ASD
Group 1: disease free >12 months adjuvant to recurrence $\widehat{\Delta}=-0.05\left(-0.30,0.20\right)$			
	*n* = 35	*n* = 38	
Age (years)	64.6 (±5.6)	65.0 (±5.1)	0.08
Stage 4			
No	1 (2.9%)	0 (0.0%)	0.24
Yes	34 (97.1%)	38 (100.0%)	0.24
Site			
Viscereal	19 (54.3%)	15 (39.5%)	0.3
Bone only	5 (14.3%)	6 (15.8%)	0.04
Other	11 (31.4%)	17 (44.7%)	0.28
Previous treatment			
None	24 (68.6%)	19 (50.0%)	0.38
Chemo only	6 (17.1%)	8 (21.1%)	0.1
Hormonal only	3 (8.6%)	4 (10.5%)	0.07
Chemo + hormonal	2 (5.7%)	7 (18.4%)	0.4
ECOG score			
0	15 (42.9%)	14 (36.8%)	0.12
1	20 (57.1%)	24 (63.2%)	0.12
Disease free >12 months adjuvant to recurrence			
Yes	35 (100.0%)	38 (100.0%)	0
Group 2: not disease free >12 months adjuvant to recurrence $\widehat{\Delta}=-0.35\left(-0.53,0.17\right)$			
	*n* = 65	*n* = 62	
Age (years)	65.2 (±5.9)	64.9 (±5.0)	0.06
Stage 4			
No	3 (4.6%)	1 (1.6%)	0.17
Yes	62 (95.4%)	61 (98.4%)	0.17
Site			
Visceral	34 (52.3%)	34 (54.8%)	0.05
Bone only	8 (12.3%)	7 (11.3%)	0.03
Other	23 (35.4%)	21 (33.9%)	0.03
Previous treatment			
None	32 (49.2%)	33 (53.2%)	0.08
Chemo only	11 (16.9%)	11 (17.7%)	0.02
Hormonal only	13 (20.0%)	15 (24.2%)	0.1
Chemo + hormonal	9 (13.8%)	3 (4.8%)	0.31
ECOG score			
0	30 (46.2%)	23 (37.1%)	0.18
1	35 (53.8%)	39 (62.9%)	0.18
Disease free >12 months adjuvant to recurrence			
No	65 (100.0%)	62 (100.0%)	0

*ASD* absolute standardized difference, *ECOG* Eastern Cooperative Oncology Group

**Table 5:** Simulation 1A subgroup discovery using random forest (five subgroups found)

	Single agent	Combination	ASD
Group 1: disease free >12 months adjuvant to recurrence $\widehat{\Delta}=-0.05\left(-0.30,0.20\right)$			
	*n* = 35	*n* = 38	
Age (years)	64.6 (±5.6)	65.0 (±5.1)	0.08
Stage 4			
No	1 (2.9%)	0 (0.0%)	0.24
Yes	34 (97.1%)	38 (100.0%)	0.24
Site			
Visceral	19 (54.3%)	15 (39.5%)	0.3
Bone only	5 (14.3%)	6 (15.8%)	0.04
Other	11 (31.4%)	17 (44.7%)	0.28
Previous treatment			
None	24 (68.6%)	19 (50.0%)	0.38
Chemo only	6 (17.1%)	8 (21.1%)	0.1
Hormonal only	3 (8.6%)	4 (10.5%)	0.07
Chemo + hormonal	2 (5.7%)	7 (18.4%)	0.4
ECOG score			
0	15 (42.9%)	14 (36.8%)	0.12
1	20 (57.1%)	24 (63.2%)	0.12
Disease free >12 months adjuvant to recurrence			
Yes	35 (100.0%)	38 (100.0%)	0
Group 2: not disease free >12 months adjuvant to recurrence, previous treatment hormonal or chemo + hormonal, site bone only or other $\widehat{\Delta}=0.06\left(-0.40,0.53\right)$			
	*n* = 11	*n* = 10	
Age (years)	63.9 (±4.9)	63.6 (±3.4)	0.07
Stage 4			
Yes	11 (100.0%)	10 (100.0%)	NaN
Site			
Bone only	2 (18.2%)	0 (0.0%)	0.67
Other	9 (81.8%)	10 (100.0%)	0.67
Previous treatment			
Hormonal only	6 (54.5%)	9 (90.0%)	0.86
Chemo + hormonal	5 (45.5%)	1 (10.0%)	0.86
ECOG score			
0	4 (36.4%)	5 (50.0%)	0.28
1	7 (63.6%)	5 (50.0%)	0.28
Disease free >12 months adjuvant to recurrence			
No	11 (100.0%)	10 (100.0%)	0
Group 3: not disease free >12 months adjuvant to recurrence, previous treatment hormonal or chemo + hormonal, site visceral $\widehat{\Delta}=-0.57\left(-1.00,-0.08\right)$			
	*n* = 11	*n* = 8	
Age (years)	67.3 (±5.2)	64.2 (±4.8)	0.62
Stage 4			
No	2 (18.2%)	0 (0.0%)	0.67
Yes	9 (81.8%)	8 (100.0%)	0.67
Site			
Visceral	11 (100.0%)	8 (100.0%)	NaN
Previous treatment			
Hormonal only	7 (63.6%)	6 (75.0%)	0.25
Chemo + hormonal	4 (36.4%)	2 (25.0%)	0.25
ECOG score			
0	6 (54.5%)	2 (25.0%)	0.63
1	5 (45.5%)	6 (75.0%)	0.63
Disease free >12 months adjuvant to recurrence			
No	11 (100.0%)	8 (100.0%)	0
Group 4: not disease free >12 months adjuvant to recurrence, previous treatment none or chemo only, ECOG = 0 $\widehat{\Delta}=-0.10\left(-0.48,0.28\right)$			
	*n* = 20	*n* = 16	
Age (years)	63.7 (±6.4)	63.1 (±5.7)	0.1
Stage 4			
No	1 (5.0%)	0 (0.0%)	0.32
Yes	19 (95.0%)	16 (100.0%)	0.32
Site			
Visceral	11 (55.0%)	10 (62.5%)	0.15
Bone only	4 (20.0%)	2 (12.5%)	0.2
Other	5 (25.0%)	4 (25.0%)	0
Previous treatment			
None	15 (75.0%)	8 (50.0%)	0.53
Chemo only	5 (25.0%)	8 (50.0%)	0.53
ECOG score			
0	20 (100.0%)	16 (100.0%)	0
Disease free >12 months adjuvant to recurrence			
No	20 (100.0%)	16 (100.0%)	0
Group 5: not disease free >12 months adjuvant to recurrence, previous treatment none or chemo only, ECOG = 1 $\widehat{\Delta}=-0.62\left(-0.87,-0.37\right)$			
	*n* = 23	*n* = 28	
Age (years)	66.1 (±6.2)	66.5 (±4.8)	0.07
Stage 4			
No	0 (0.0%)	1 (3.6%)	0.27
Yes	23 (100.0%)	27 (96.4%)	0.27
Site			
Visceral	12 (52.2%)	16 (57.1%)	0.1
Bone only	2 (8.7%)	5 (17.9%)	0.27
Other	9 (39.1%)	7 (25.0%)	0.31
Previous treatment			
None	17 (73.9%)	25 (89.3%)	0.4
Chemo only	6 (26.1%)	3 (10.7%)	0.4
ECOG score			
1	23 (100.0%)	28 (100.0%)	0
Disease free >12 months adjuvant to recurrence			
No	23 (100.0%)	28 (100.0%)	0

*ASD* absolute standardized difference, *ECOG* Eastern Cooperative Oncology Group

**Table 6:** Simulation 1A subgroup discovery using gradient random forest (five subgroups found)

	Single agent	Combination	ASD
Group 1: not disease free >12 months adjuvant to recurrence $\widehat{\Delta}=-0.05\left(-0.30,0.20\right)$			
	*n* = 35	*n* = 38	
Age (years)	64.6 (±5.6)	65.0 (±5.1)	0.08
Stage 4			
No	1 (2.9%)	0 (0.0%)	0.24
Yes	34 (97.1%)	38 (100.0%)	0.24
Site			
Visceral	19 (54.3%)	15 (39.5%)	0.3
Bone only	5 (14.3%)	6 (15.8%)	0.04
Other	11 (31.4%)	17 (44.7%)	0.28
Previous treatment			
None	24 (68.6%)	19 (50.0%)	0.38
Chemo only	6 (17.1%)	8 (21.1%)	0.1
Hormonal only	3 (8.6%)	4 (10.5%)	0.07
Chemo + hormonal	2 (5.7%)	7 (18.4%)	0.4
ECOG score			
0	15 (42.9%)	14 (36.8%)	0.12
1	20 (57.1%)	24 (63.2%)	0.12
Disease free >12 months adjuvant to recurrence			
Yes	35 (100.0%)	38 (100.0%)	0
Group 2: not disease free >12 months adjuvant to recurrence $\widehat{\Delta}=-0.01\left(-0.42,0.39\right)$			
	*n* = 11	*n* = 15	
Age (years)	63.9 (±4.9)	64.2 (±4.1)	0.06
Stage 4			
Yes	11 (100.0%)	15 (100.0%)	0
Site			
Other	11 (100.0%)	15 (100.0%)	0
Previous treatment			
Chemo only	2 (18.2%)	5 (33.3%)	0.35
Hormonal only	5 (45.5%)	9 (60.0%)	0.29
Chemo + hormonal	4 (36.4%)	1 (6.7%)	0.78
ECOG score			
0	3 (27.3%)	8 (53.3%)	0.55
1	8 (72.7%)	7 (46.7%)	0.55
Disease free >12 months adjuvant to recurrence			
No	11 (100.0%)	15 (100.0%)	0
Group 3: not disease free >12 months adjuvant to recurrence $\widehat{\Delta}=-0.33\left(-0.92,0.25\right)$			
	*n* = 12	*n* = 6	
Age (years)	65.7 (±7.6)	65.0 (±4.4)	0.12
Stage 4			
Yes	12 (100.0%)	6 (100.0%)	0
Site			
Other	12 (100.0%)	6 (100.0%)	0
Previous treatment			
None	12 (100.0%)	6 (100.0%)	0
ECOG score			
0	5 (41.7%)	1 (16.7%)	0.57
1	7 (58.3%)	5 (83.3%)	0.57
Disease free >12 months adjuvant to recurrence			
No	12 (100.0%)	6 (100.0%)	0
Group 4: not disease free >12 months adjuvant to recurrence $\widehat{\Delta}=-0.23\left(-0.61,0.15\right)$			
	*n* = 22	*n* = 14	
Age (years)	63.7 (±6.2)	62.3 (±5.1)	0.24
Stage 4			
No	1 (4.5%)	0 (0.0%)	0.31
Yes	21 (95.5%)	14 (100.0%)	0.31
Site			
Visceral	17 (77.3%)	12 (85.7%)	0.22
Bone only	5 (22.7%)	2 (14.3%)	0.22
Previous treatment			
None	10 (45.5%)	7 (50.0%)	0.09
Chemo only	5 (22.7%)	5 (35.7%)	0.29
Hormonal only	4 (18.2%)	1 (7.1%)	0.34
Chemo + hormonal	3 (13.6%)	1 (7.1%)	0.21
ECOG score			
0	22 (100.0%)	14 (100.0%)	0
Disease free >12 months adjuvant to recurrence			
No	22 (100.0%)	14 (100.0%)	0
Group 5: not disease free >12 months adjuvant to recurrence $\widehat{\Delta}=-0.64\left(-0.90,-0.39\right)$			
	*n* = 20	*n* = 27	
Age (years)	67.3 (±4.5)	66.5 (±5.1)	0.15
Stage 4			
No	2 (10.0%)	1 (3.7%)	0.25
Yes	18 (90.0%)	26 (96.3%)	0.25
Site			
Visceral	17 (85.0%)	22 (81.5%)	0.09
Bone only	3 (15.0%)	5 (18.5%)	0.09
Previous treatment			
None	10 (50.0%)	20 (74.1%)	0.51
Chemo only	4 (20.0%)	1 (3.7%)	0.52
Hormonal only	4 (20.0%)	5 (18.5%)	0.04
Chemo + hormonal	2 (10.0%)	1 (3.7%)	0.25
ECOG score			
1	20 (100.0%)	27 (100.0%)	0
Disease free >12 months adjuvant to recurrence			
No	20 (100.0%)	27 (100.0%)	0

*ASD* absolute standardized difference, *ECOG* Eastern Cooperative Oncology Group

**Table 7:** Data at randomization for simulation 1B

	Single agent	Combination	ASD
	*n* = 100	*n* = 100	
Age (years)	63.8 (±5.0)	64.3 (±5.5)	0.1
Stage 4			
No	1 (1.0%)	0 (0.0%)	0.14
Yes	99 (99.0%)	100 (100.0%)	0.14
Site			
Visceral	42 (42.0%)	46 (46.0%)	0.08
Bone only	18 (18.0%)	15 (15.0%)	0.08
Other	40 (40.0%)	39 (39.0%)	0.02
Previous treatment			
None	55 (55.0%)	44 (44.0%)	0.22
Chemo only	18 (18.0%)	18 (18.0%)	0
Hormonal only	19 (19.0%)	24 (24.0%)	0.12
Chemo + hormonal	8 (8.0%)	14 (14.0%)	0.19
ECOG score			
0	46 (46.0%)	44 (44.0%)	0.04
1	54 (54.0%)	56 (56.0%)	0.04
Disease free >12 months adjuvant to recurrence			
No	73 (73.0%)	71 (71.0%)	0.04
Yes	27 (27.0%)	29 (29.0%)	0.04

*ASD* absolute standardized difference, *ECOG* Eastern Cooperative Oncology Group

**Table 8:** Data at randomization for simulation 2B

	Placebo	Treatment	ASD
	*n* = 3000	*n* = 3000	
Age (years)	68.1 (±10.1)	68.1 (±10.0)	0.01
Black race			
No	2,090 (69.7%)	2,154 (71.8%)	0.05
Yes	910 (30.3%)	846 (28.2%)	0.05
Systolic blood pressure (mm Hg)	140.3 (±15.1)	140.1 (±14.7)	0.01
Diastolic blood pressure (mm Hg)	78.2 (±12.1)	77.6 (±12.1)	0.05
Serum creatinine (mg/dl)	1.1 (±0.3)	1.1 (±0.3)	0
eGFR (ml min^–1^ 1.73 m^–2^)	71.6 (±20.3)	71.6 (±19.7)	0
Statin use			
No	1,748 (58.3%)	1,718 (57.3%)	0.02
Yes	1,252 (41.7%)	1,282 (42.7%)	0.02
Aspirin use			
No	1,512 (50.4%)	1,439 (48.0%)	0.05
Yes	1,488 (49.6%)	1,561 (52.0%)	0.05
Framingham risk score	25.2 (±11.9)	25.0 (±11.9)	0.02
Smoking status			
Never	1,303 (43.4%)	1,324 (44.1%)	0.01
Former	1,301 (43.4%)	1,242 (41.4%)	0.04
Current	396 (13.2%)	434 (14.5%)	0.04

*ASD* absolute standardized difference, *eGFR* estimated glomerular filtration rate

**Table 9:** Data at randomization for simulation 2C

	Placebo	Treatment	ASD
	*n* = 3000	*n* = 3000	
Age (years)	68.0 (±9.8)	67.8 (±10.1)	0.03
Black race			
No	2,096 (69.9%)	2,089 (69.6%)	0.01
Yes	904 (30.1%)	911 (30.4%)	0.01
Systolic blood pressure (mm Hg)	140.0 (±15.1)	139.2 (±14.9)	0.05
Diastolic blood pressure (mm Hg)	78.4 (±12.0)	77.4 (±11.9)	0.08
Serum creatinine (mg/dl)	1.1 (±0.3)	1.1 (±0.3)	0.01
eGFR (ml min^–1^ 1.73 m^–2^)	71.7 (±19.6)	72.2 (±20.2)	0.02
Statin use			
No	1,692 (56.4%)	1,678 (55.9%)	0.01
Yes	1,308 (43.6%)	1,322 (44.1%)	0.01
Aspirin use			
No	1,518 (50.6%)	1,467 (48.9%)	0.03
Yes	1,482 (49.4%)	1,533 (51.1%)	0.03
Framingham risk score	24.7 (±11.9)	25.1 (±11.6)	0.04
Smoking status			
Never	1,334 (44.5%)	1,312 (43.7%)	0.01
Former	1,254 (41.8%)	1,256 (41.9%)	0
Current	412 (13.7%)	432 (14.4%)	0.02

*ASD* absolute standardized difference, *eGFR* glomerular filtration rate

## Abbreviations

ASD: Absolute standardized difference
ATE: Average treatment effect
CVD: Cardiovascular disease
ECOG: Eastern Cooperative Oncology Group
eGFR: Estimated glomerular filtration rate
HTE: Heterogeneous treatment effect
LR: Logistic regression
mCART: Matching plus classification and regression trees
RF: random forest
